# Testing bias in clinical databases: methodological considerations

**DOI:** 10.1186/1742-7622-7-2

**Published:** 2010-05-14

**Authors:** Karin J Velthove, Hubert GM Leufkens, Patrick C Souverein, René C Schweizer, Wouter W van Solinge

**Affiliations:** 1 Faculty of Science, Division of Pharmacoepidemiology and Pharmacotherapy, Utrecht Institute of Pharmaceutical Sciences, Utrecht University, The Netherlands; 2 Department of Clinical Chemistry and Haematology, University Medical Center Utrecht, Utrecht, The Netherlands; 3 Department of Respiratory Medicine, Division of Heart and Lungs, University Medical Center Utrecht, Utrecht, The Netherlands

## Abstract

**Background:**

Laboratory testing in clinical practice is never a random process. In this study we evaluated testing bias for neutrophil counts in clinical practice by using results from requested and non-requested hematological blood tests.

**Methods:**

This study was conducted using data from the Utrecht Patient Oriented Database. This clinical database is unique, as it contains physician requested data, but also data that are not requested by the physician, but measured as result of requesting other hematological parameters. We identified adult patients, hospitalized in 2005 with at least two blood tests during admission, where requests for general blood profiles and specifically for neutrophil counts were contrasted in scenario analyses. Possible effect modifiers were diagnosis and glucocorticoid use.

**Results:**

A total of 567 patients with requested neutrophil counts and 1,439 patients with non-requested neutrophil counts were analyzed. The absolute neutrophil count at admission differed with a mean of 7.4 × 10^9^/l for requested counts and 8.3 × 10^9^/l for non-requested counts (p-value < 0.001). This difference could be explained for 83.2% by the occurrence of cardiovascular disease as underlying disease and for 4.5% by glucocorticoid use.

**Conclusion:**

Requests for neutrophil counts in clinical databases are associated with underlying disease and with cardiovascular disease in particular. The results from our study show the importance of evaluating testing bias in epidemiological studies obtaining data from clinical databases.

## Background

In recent years, large health care databases are increasingly used and provide important tools in epidemiological research [1,2]. Advantages are that large amounts of clinical data are available at relatively low cost, and that these databases usually reflect daily practice [3,4]. However, in contrast to randomized clinical trials, where data collection is well-controlled, bias should always be considered when using routinely collected data in automated databases and methodological issues should be taken into account [3,5-7].

Laboratory testing in clinical practice is never a random process, as the physician has reasons to perform a test. Physicians selectively request tests for patients with a high probability of abnormalities and less frequently for patients with a low probability, because of patient burden and costs [8]. Such selective processes might induce testing bias in clinical database studies. There are several strategies to minimize testing bias, including selection of proper patient populations, measuring outcomes for all study participants, blind testing, or using imputation techniques to deal with missing data [8-10], but these techniques do not provide insight into size and direction of testing bias.

One example where testing bias might occur is in physicians' requests of blood tests. Neutrophil counts in peripheral blood are considered a useful biomarker for disease severity in many conditions [11-14]. However, testing bias might occur because of underlying disease or medication use, as neutrophil counts differ in several diseases and clinical observations have shown that patients using glucocorticoids often have higher neutrophil counts. Requesting neutrophil counts specifically for certain diseases or for glucocorticoid users might cause testing bias in clinical databases. The aim of this study was to evaluate testing bias for neutrophil counts in clinical practice by using results from requested and non-requested hematological blood tests.

## Methods

### Setting

This study was conducted using data from the Utrecht Patient Oriented Database (UPOD). UPOD is an infrastructure of relational databases comprising administrative data on patient characteristics, laboratory test results, medication orders, discharge diagnoses and medical procedures for all patients treated at the University Medical Center (UMC) Utrecht, a 1,042-bed tertiary teaching hospital in the center of the Netherlands. Each year, approximately 165,000 patients are treated during more than 28,000 hospitalizations, 15,000 day-care treatments, and 334,000 outpatient visits. UPOD data acquisition and data management is in accordance with current Dutch privacy and ethical regulations. A more complete description of UPOD has been published elsewhere [15].

**Figure 1:**
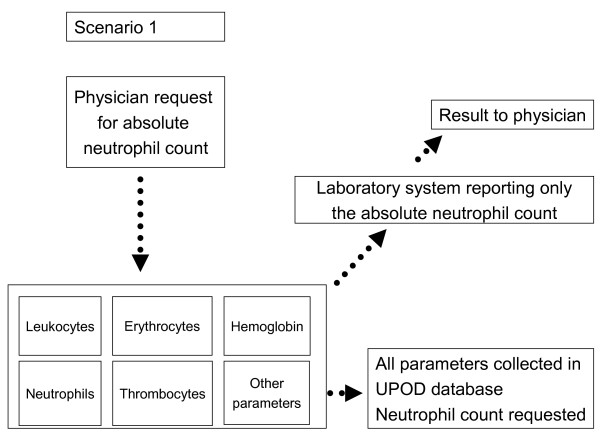
**Illustration of scenarios**. Scenario 1 represents the situation in a typical clinical database where all neutrophil counts were requested by physicians.

**Figure 2:**
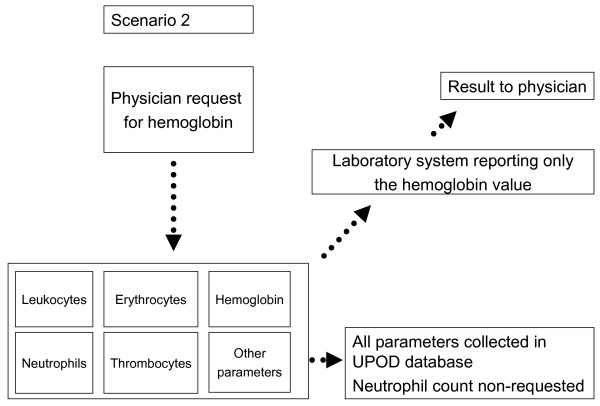
**Is unique for UPOD as this includes non-requested neutrophil counts**. These non-requested neutrophil counts are measured because this test is conducted automatically when one hematological test, for example hemoglobin, is requested.

UPOD is a unique clinical database as it contains results of hematological blood tests measured with Cell-Dyn Sapphire automated blood cell analyzers (Abbott Diagnostics, St. Clara, California, USA) [15]. A feature of this analyzer is that it measures all hematological parameters irrespective of whether these are requested or not [15]. The non-requested parameters are measured because one hematological test is technically linked to the other hematological tests and conducted automatically when one of these tests is requested. In other words, UPOD contains requested (Figure 1) and non-requested test results (Figure 2). Although non-requested neutrophil counts are not reported to the clinician, these neutrophil counts are collected in UPOD.

### Scenarios

By comparing the measured hematological parameters with the routine hospital laboratory reporting system, which reports laboratory results to physicians, neutrophil counts can be categorized as requested or non-requested. Neutrophil counts appearing in the laboratory reporting system were categorized as requested; other neutrophil counts were categorized as non-requested. Using these data, we conducted two scenario analyses. Scenario 1 reflects the situation as in a typical clinical database, where all blood tests were requested. With scenario 2 we were able to study testing bias by including non-requested blood tests in our analysis.

### Study population

The source population comprised 3,467 adult (18 years or older) users and non-users of glucocorticoids who were hospitalized in the UMC Utrecht in 2005 and had at least two hematological blood tests, where these tests should cover at least a one-day period. For each glucocorticoid user, the first blood test during admission and the last blood measurement during in-hospital glucocorticoid use were selected for analysis. Up to four unexposed patients were sampled to each glucocorticoid user according to calendar time (with a maximum of 15 days before or after the test date of the user), neutrophil count at time of admission (max 2 × 10^9 ^neutrophils/l around the neutrophil count of the user) and days between the two blood samples (max two days around the number of days for the glucocorticoid user). According to our laboratory normal reference range for neutrophils (1.6-8.3 × 10^9^/l), there is large inter-individual variation in the absolute neutrophil count. Using two blood tests, we were able to study testing bias in both blood tests separately, but also in the change in neutrophil count during hospitalization for each patient. Within the source population, we contrasted patients with both blood tests requested and with both blood tests non-requested, one at time of admission and one at the end of hospitalization. For all participants the discharge diagnosis was defined according to the ICD-9-CM code [16].

### Data analysis

Student t-tests, Mann-Whitney tests, and chi-square tests were used to test for differences between groups, as appropriate. Confounding was studied using logistic regression. The absolute neutrophil count was categorized into tertiles to obtain three equally-sized groups. These three groups were defined as an increase, decrease or no change in neutrophil count where no change was the comparator group. Potential confounding factors were included in a multivariable logistic model in sequence with decreasing confounding strength. Potential confounders that were studied were age, gender, number of days between blood samples, length of hospitalization, death during hospitalization, and diagnosis. All variables that changed the regression coefficient for glucocorticoid use by less than ten percent were excluded from the model. Of these potential confounders, only diagnosis had a substantial effect on the comparison between scenarios. Glucocorticoid use was studied because of the ongoing discussion about the effect of glucocorticoids on the neutrophil count [12,17,18]. Subsequently, linear regression analysis was used to estimate the proportion of bias associated with diagnostic subgroups and glucocorticoid use. The beta-coefficient for the contrasted scenarios was calculated for all patients in the study population as well as for only patients exposed to one factor (for example a diagnostic subgroup or glucocorticoid use). The proportion of bias explained by one factor was calculated as the weighted fraction of beta-coefficients. All analyses were conducted using SPSS for Windows, version 14.0 (SPSS Inc., Chicago, Illinois, USA).

## Results

A total of 567 patients with requests for the absolute neutrophil count (scenario 1) and 1,439 patients with non-requested neutrophil counts (scenario 2) were identified. It appeared that the absolute neutrophil count was most frequently requested in the context of a leukocyte differential request, which includes the absolute counts of neutrophils, eosinophils, lymphocytes, monocytes, and basophils (99.8% of all neutrophil count requests). Of patients with requested neutrophil counts, there was also a hemoglobin request for 97.2% of patients. For patients with non-requested neutrophil counts, 96.1% of the requests were for hemoglobin. Hemoglobin values were lower when requested compared with non-requested hemoglobin values (Table 1).

**Table 1:** Baseline characteristics of the study population*

Characteristic	Requested neutrophil count (scenario 1)(n = 567)	Non-requested neutrophil count (scenario 2)(n = 1,439)	P-value (two-sided)
Age, yr	57.8 ± 17.8	56.9 ± 19.0	0.311^a^
Sex			0.001^c^
Male	327 (57.7)	715 (49.7)	
Female	240 (42.3)	724 (50.3)	
Length of hospitalization	10 (7-18)	10 (6-18)	0.807^b^
Days between blood tests	6 (3-10)	4 (2-8)	< 0.001^b^
Death during hospitalization	36 (6.3)	83 (5.8)	0.610^c^

Hemoglobin value 1^st ^blood test (mmol/l)	8.0 ± 1.3	7.4 ± 1.4	< 0.001^b^
Median (IQR)	8.1 (7.2-8.9)	7.4 (6.4-8.4)	
Hemoglobin value 2^nd ^blood test (mmol/l)	7.1 ± 1.3	6.9 ± 1.2	< 0.001^b^
Median (IQR)	7.1 (6.2-8.0)	6.8 (5.9-7.7)	

Absolute neutrophil count 1^st ^blood test (10^9^/l)	7.4 ± 4.7	8.3 ± 4.0	< 0.001^b^
Median (IQR)	6.1 (4.0-9.9)	7.7 (5.3-10.6)	
Neutropenia	17 (3.0)	17 (1.2)	
Within normal reference area	365 (64.4)	784 (54.5)	
Neutrophilia	185 (32.6)	638 (44.3)	< 0.001^c^
Absolute neutrophil count 2^nd ^blood test (10^9^/l)	7.6 ± 4.7	7.8 ± 4.0	0.111^b^
Median (IQR)	6.8 (4.5-9.5)	7.0 (5.0-9.5)	
Neutropenia	20 (3.5)	18 (1.3)	0.003^c^
Within normal reference area	352 (62.1)	915 (63.6)	
Neutrophilia	195 (34.4)	506 (35.2)	

Change in neutrophil count for each patient (10^9^/l)	0.14 ± 5.1	-0.50 ± 4.2	0.008^a^

Glucocorticoid use	174 (30.7)	237 (16.5)	< 0.001^c^
Diagnosis			< 0.001^c^
Neoplasms	71 (12.5)	220 (15.3)	
Cardiovascular diseases	200 (35.3)	380(26.4)	
Respiratory diseases	53 (9.3)	38 (2.6)	
Infectious and parasitic diseases	30 (5.3)	21 (1.5)	
Endocrine, nutritional and metabolicdiseases, and immunity disorders	30 (5.3)	22 (1.5)	
Diseases of the digestive system	26 (4.6)	114 (7.9)	
Diseases of the genitourinary system	33 (5.8)	49 (3.4)	
Diseases of the skin, subcutaneous tissue, musculoskeletal system, and connective tissue	18 (3.2)	108 (7.5)	
Other	106 (18.7)	487 (33.8)	

*Data are presented as n (%), or mean ± SD, or median (interquartile range)

^a^Student t-test; ^b^Mann-Whitney test; ^c^*χ*^2 ^test

Scenario 1 includes patients with requested neutrophil counts for both blood tests

Scenario 2 includes patients with non-requested neutrophil counts for both blood tests

**Figure 3:**
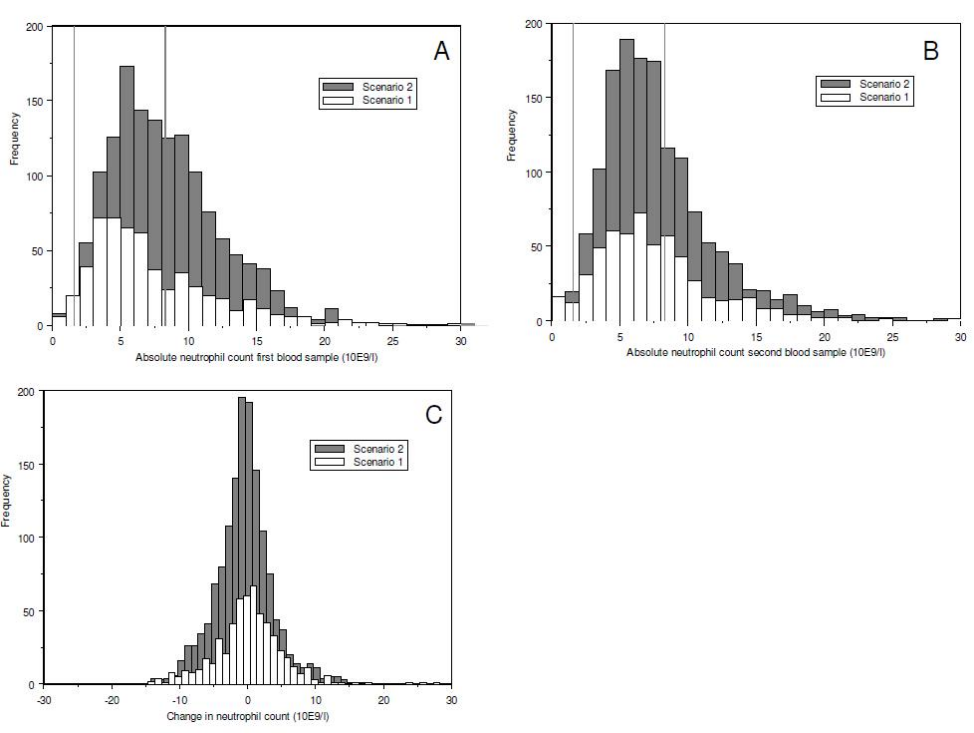
**Absolute neutrophil counts and change in neutrophil count**. Distribution of the absolute neutrophil count of the first (A) and second blood test (B) and the change in neutrophil count for each patient (C) for requested neutrophil counts (scenario 1) and non-requested neutrophil counts (scenario 2). The vertical lines represent the normal reference area of 1.6-8.3 × 10^9^/l for the absolute neutrophil count (A and B).

For the first blood test, lower neutrophil counts were found for patients with requested neutrophil counts compared with non-requested neutrophil counts (Table 1). Comparable neutrophil counts were found in the second blood test. For both blood tests, there were more patients with neutropenia and fewer patients with neutrophilia for requested neutrophil counts. Studying the change in the absolute neutrophil count during hospitalization for each patient, patients with non-requested neutrophil counts had a mean decrease of 0.50 × 10^9 ^neutrophils/l compared with a slight increase of 0.14 × 10^9 ^neutrophils/l for patients with requested neutrophil counts (p-value 0.008, Table 1, Figure 3).

**Figure 4:**
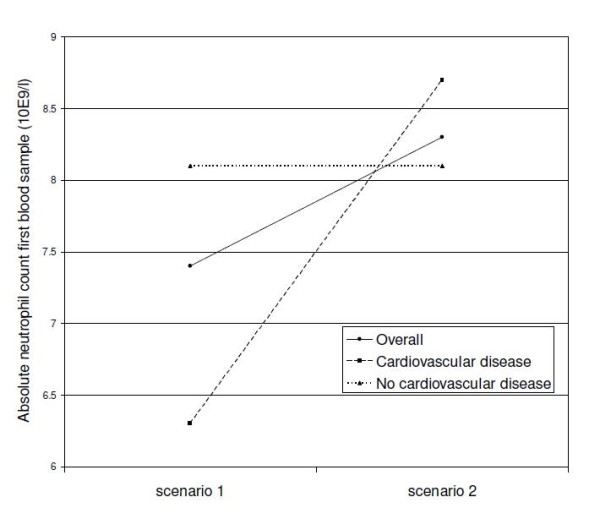
**The effect of cardiovascular disease on the absolute neutrophil count of the first blood test**. The higher neutrophil count in scenario 2 is explained by a high neutrophil count among cardiovascular patients in scenario 2. Excluding cardiovascular disease from analysis, there was no difference between the scenarios. Scenario 1 included requested neutrophil counts, scenario 2 included non-requested neutrophil counts.

Overall, the main diagnostic subgroups were cardiovascular disease (28.9%), neoplasms (14.5%), and respiratory disease (4.5%). Requests for neutrophil counts were more often conducted for patients suffering from cardiovascular or respiratory diseases, whereas diagnoses for non-requested neutrophil counts were much more diffuse with multiple diagnoses (Table 1). There were no differences in absolute neutrophil count or change in neutrophil counts among patients with neoplasms and respiratory disease (Table 2). However, among patients with cardiovascular disease there was a lower absolute neutrophil count in the first blood test for requested counts compared with non-requested neutrophil counts. Excluding cardiovascular patients from analysis, the absolute neutrophil counts in the first blood test were equal with 8.1 × 10^9^/l in both scenarios (Figure 4). The difference in absolute neutrophil count between scenarios in the first blood test could be explained for 83.2% by cardiovascular disease (p-value for effect modification 0.002). Incorporating glucocorticoid use in the linear regression model showed that diagnosis was far more important than glucocorticoid use (p-value for effect modification 0.240). Taking diagnosis into account, 4.5% of the difference in absolute neutrophil count between scenarios in the first blood test could be explained by glucocorticoid use.

**Table 2:** Neutrophil counts for the main diagnostic subgroups

	Cardiovascular disease		
**Characteristic**	**Requested (scenario 1)**	**Non-requested (scenario 2)**	**P-value (two-sided)**
**N**	**200 (35.3%)**	**380 (26.4%)**	

Absolute neutrophil count 1^st ^blood test (10^9^/l)	6.3 ± 3.6	8.7 ± 4.0	< 0.001^b^
Median (IQR)	5.3 (3.9-7.1)	8.2 (5.6-11.2)	
Absolute neutrophil count 2^nd ^blood test (10^9^/l)	8.4 ± 4.0	8.3 ± 4.0	0.741^b^
Median (IQR)	7.5 (5.5-9.8)	7.5 (5.4-10.5)	
Change in neutrophil count for each patient (10^9^/l)	2.1 ± 4.5	-0.4 ± 4.2	< 0.001^a^

	Neoplasms		

N	71 (12.5%)	220 (15.3%)	

Absolute neutrophil count 1^st ^blood test (10^9^/l)	6.6 ± 4.1	7.1 ± 3.6	0.284^b^
Median (IQR)	6.0 (3.0-10.2)	6.5 (4.6-9.4)	
Absolute neutrophil count 2^nd ^blood test (10^9^/l)	7.7 ± 5.6	7.5 ± 4.3	0.986^b^
Median (IQR)	6.9 (4.1-10.6)	6.9 (4.8-9.0)	
Change in neutrophil count for each patient (10^9^/l)	1.1 ± 5.4	0.5 ± 4.2	0.348^a^

	Respiratory disease		
N	53 (9.3%)	38 (2.6%)	

Absolute neutrophil count 1^st ^blood test (10^9^/l)	10.2 ± 6.0	9.3 ± 4.1	0.646^b^
Median (IQR)	9.2 (6.3-12.8)	8.7 (6.2-12.0)	
Absolute neutrophil count 2^nd ^blood test (10^9^/l)	8.9 ± 7.4	8.0 ± 4.0	0.778^b^
Median (IQR)	7.7 (5.6-9.8)	7.5 (5.5-9.2)	
Change in neutrophil count for each patient (10^9^/l)	-1.3 ± 6.8	-1.3 ± 4.3	0.991^a^

*Data are presented as n (%), mean ± SD, or median and interquartile range

^a ^Student t-test; ^b ^Mann-Whitney test

Scenario 1 includes patients with requested neutrophil counts for both blood tests

Scenario 2 includes patients with non-requested neutrophil counts for both blood tests

With respect to the absolute neutrophil count in the second blood test, there were no differences between the scenarios, either in the overall analysis or in the main diagnostic subgroups. An increase in neutrophil count of 2.1 × 10^9^/l was shown for requested neutrophil counts in cardiovascular patients, whereas non-requested counts revealed a decrease of 0.4 × 10^9 ^neutrophils/l (Table 2). Excluding cardiovascular disease from analysis, the change in neutrophil count was comparable in both scenarios with a mean decrease of 0.9 × 10^9^/l for each patient with requested neutrophil counts and a mean decrease of 0.5 × 10^9^/l for each patient including only non-requested neutrophil counts (p-value = 0.211).

## Discussion

In this study, we used UPOD to study bias in neutrophil testing, as this database contains both requested and non-requested neutrophil counts. Among tests for which neutrophil counts were requested, hemoglobin was also requested in 97%. For non-requested neutrophil test results, 96% were generated by hemoglobin requests. Therefore, hemoglobin requests approximate random testing and can be used as comparator group. Absolute neutrophil counts differed for requested tests (scenario 1) compared with non-requested tests (scenario 2), which leads to the conclusion that testing bias was found in this study.

The bias in absolute neutrophil count in the first blood test could be explained for 83.2% by cardiovascular disease. This finding could reflect the role of neutrophils in cardiovascular disease [13,19,20]. After excluding cardiovascular disease from analysis, there were no differences in absolute neutrophil count or change in neutrophil count for each patient. This could be explained by the fact that the absolute neutrophil count was mainly requested in the context of a leukocyte differential count. Similar findings were observed for the change in neutrophil count. For the second neutrophil test, no differences were found between the scenarios. The second neutrophil test was at the end of hospitalization. The first neutrophil test, at time of admission, is more informative because the patients are likely to be severely ill at that point. At time of the second blood test the difference in absolute neutrophil count has evened out, as patients are healthier at the end of hospitalization.

The results of this study are in accordance with other studies finding that data are not missing at random [21,22]. Further research is needed to study the clinical relevance of the bias found in this study. Distributions of diagnostic subgroups and testing guidelines might vary between health care institutions. As a consequence, generalizability of clinical implications, like the association with cardiovascular disease as an example in this study, might be limited. However, testing bias is an issue in all centers and should be evaluated to be able to adjust for this bias. Using laboratory tests, a random tested parameter, like hemoglobin testing in this study, could serve as comparator group to study testing bias. With knowledge about the size and direction of testing bias, strategies such as imputation techniques [8,21] could adjust for this bias in order to obtain an unbiased risk estimate in epidemiological studies.

With development of automated machines for routine analysis, more parameters are measured than requested. When these non-requested parameters are collected, testing randomness is introduced. UPOD contains requested neutrophil counts and non-requested neutrophil counts, as well as other non-requested hematological blood tests. Therefore, the database is especially suitable to study and adjust for testing bias in clinical research questions. Conducting studies with laboratory markers in UPOD, correction factors for requested testing can be added to the statistical model to minimize testing bias in order to obtain an unbiased risk estimate.

A classic example of testing bias is the association between thrombosis and use of oral contraceptives. Many studies state traditionally that the size of this association is overestimated because of diagnostic suspicion bias and referral bias, both types of testing bias [23,24]. However, a case-control study with the same referral and diagnostic strategies for cases and controls, showed that neither type of bias played a major role in previous studies, and that the risk of thrombosis while using oral contraceptives is not solely due to bias [9]. This example and the results from our study show the importance of evaluating testing bias in epidemiological studies obtaining data from clinical databases.

## List of abbreviations

UMC: University Medical Center; UPOD: Utrecht Patient Oriented Database.

## Competing interests

None of the authors report a personal conflict of interest. The division of Pharmacoepidemiology & Pharmacotherapy employing authors KJV, HGML, PCS, and WWS has received unrestricted funding for pharmacoepidemiological research from GlaxoSmithKline, Novo Nordisk, the private-public funded Top Institute Pharma (http://www.tipharma.nl, includes co-funding from universities, government, and industry), the Dutch Medicines Evaluation Board, and the Dutch Ministry of Health.

## Authors' contributions

KJV participated in designing the study, performed the analyses and drafted the final manuscript. HGML, PCS, RCS and WWS participated in the design of the study and helped in the interpretation of the data. All authors critically reviewed draft versions of the manuscript. All authors read and approved the final manuscript.
